# Diel Rhythmicity of Field Responses to Synthetic Pheromone Lures in the Pine Sawyer *Monochamus saltuarius*

**DOI:** 10.3390/insects12050441

**Published:** 2021-05-12

**Authors:** Junheon Kim, Younghak Jung, Sangmyeong Lee

**Affiliations:** 1 Forest Insect Pests and Diseases Division, National Institute of Forest Science, Seoul 02455, Korea; 2 SM Biovision Co., Jinju, Gyeongnam 52849, Korea; gkr315@naver.com (Y.H.J.); lsm1918@hanmail.net (S.-M.L.)

**Keywords:** longhorn beetles, behavior, aggregation pheromone, attractant, pine wilt disease

## Abstract

**Simple Summary:**

*Monochamus saltuarius* is a vector of pine wood nematode (PWN) in Korea and eastern China. The diel rhythmicity of *M. saltuarius* in response to an aggregation pheromone and attractants (PA) was studied with the aid of a spray device controlled with an electronic timer. Our study revealed that the flight activity of *M. saltuarius* in response to PA was diurnal. The results improve the understanding of the behavioral biology of *M. saltuarius*, allowing the development of pest management strategies to prevent the spread of PWN and control its vector.

**Abstract:**

The pine wood nematode (PWN), *Bursaphelenchus xylophilus*, causes lethal pine wilt disease (PWD) in Asia and Europe and has become a serious threat to global pine forest ecosystems. In Korea, *Monochamus saltuarius* transmits PWN not only to *Pinus densiflora*, but also to *Pinus koraiensis*, which is widely distributed across eastern Asia. The diel rhythmicity of *M. saltuarius* in response to its aggregation pheromone was studied with the aim of providing reliable data for the prevention of PWD and control of *Monochamus* spp. Using a spray dispenser controlled with an electronic timer, *M. saltuarius* pheromone and attractants (PA) were sprayed to determine the diel rhythm of the response to PA. The spraying period was divided into four time periods: 05:00–11:00 (time period A), 11:00–17:00 (time period B), 17:00–23:00 (time period C), and 23:00–05:00 (time period D). The largest number of *M. saltuarius* was caught in time period B, followed by A, C, and D. It could be concluded that the flight activity of *M. saltuarius* in response to PA was diurnal. The results of this study improve the understanding of the behavioral biology of *M. saltuarius*, allowing for the development of pest management strategies to prevent the spread of PWN and control its vector.

## 1. Introduction

The pine wood nematode (PWN), *Bursaphelenchus xylophilus* (Steiner and Buhrer) Nicke, is a plant parasitic nematode that causes lethal pine wilt disease (PWD) in Asia and Europe. This epidemic disease has become a serious threat to global pine forest ecosystems [1]. Important vectors of PWN including various species of *Monochamus* beetles (Coleoptera: Cerambycidae) have been identified [2,3,4,5]. In Korea, two beetles, *Monochamus alternatus* and *M. saltuarius*, have been identified as vectors of PWN. Among them, *M. saltuarius* transmits PWN not only to *Pinus densiflora*, but also to Korean pine, *P. koraiensis* [6]. *P. koraiensis* is widely distributed across eastern Asia including Korea, China, Japan, and Siberia [7]. In 2017, thousands of Korean pines that had died due to PWD were reported in northeastern China, and it was revealed that PWD was caused by PWN, which was vectored by *M. saltuarius* in northeast China [8]. *M. saltuarius* emerged from *P. tabuliformis* carried pine wood nematode and its carrier rate reached 58.3%, and the average carrying capacity of *M. saltuarius* of pine wood nematodes was 642.4 [9].

*M. saltuarius* is distributed across eastern Asia and Europe [10]. Biological characteristics such as reproductive traits, larval diapause time, sex ratio, and oviposition and mating behavior of *M. saltuarius* are known to be very similar to those of *M. alternatus* [11,12,13], though the body size of *M. saltuarius* is somewhat smaller than that of *M. alternatus* [10]. *M. saltuarius* is generally distributed in cold regions, while *M. alternatus* is distributed in warmer climate regions. The habitats of *M. saltuarius* and *M. alternatus* overlap in some regions in Korea [14].

The behavioral patterns of *M. alternatus* such as movement, mating, and reproductive behaviors are well studied in Korea and Japan [15,16,17,18,19]. Since *M. saltuarius* is a known vector of PWN in Korea, the life cycle and mating behavior have been studied [12,13]; recently, dispersal capacity using flight mills has also been studied [20].

Since monochamol, 1-(2-undecyloxy)-ethanol, was first reported as an aggregation pheromone of *Monochamus galloprovincialis*, which is a vector of PWN in Europe [5], monochamol has been identified as a common component of the aggregation pheromone of several *Monochamus* species including *M. saltuarius* [21,22,23,24]. Many studies have focused on the attractiveness of aggregation pheromones and synergists for population monitoring or large-scale trapping of *Monochamus* spp. [25,26,27,28,29]. Diel rhythm in response to monochamol and attractants in the field by *M. scutellatus scutellatus* and *M. notatus*, a close congener, has been reported [30]. Understanding the behavioral biology of *Monochamus* spp. can aid in the development of pest management strategies to prevent the spread of PWN and control its vector. We studied the diel rhythmicity of *M. saltuarius* in response to its aggregation pheromone with the aim of providing reliable data to aid in developing pest management strategies for the prevention of PWD and control of *Monochamus saltuarius*.

## 2. Materials and Methods

### 2.1. Study Site and Period

The field experiments to assess the effects of emission intervals were conducted in pine tree forest stands located in Seongpyeong (36°39′27″ N, 128°24′01″ E), Yecheon, Gyeongsangbuk-do, and those to assess the diel rhythm of the response to its aggregation pheromone and attractants (PA) were conducted in Susim (36°39′23″ N, 128°24′00″ E), Yecheon, Gyeongsangbuk-do. The forest stands were dominated by mature *P. densiflora* and *P. koraiensis*, with a ratio of *P. densiflora* to *P. koraiensis* of approximately 7:3. The experiments were performed from 21 April to 5 June, 2017. Peak adult emergence occurred from 4 May to 19 May, 2017 (Figure S1).

### 2.2. Periodicity of the Response of M. saltuarius to Pheromone

#### 2.2.1. Pheromone Source

We used a spray dispenser affixed to a black-colored cross-vane trap (Alpha Scents, Portland, OR, USA). Recently, it has been reported that the trapping efficacy can be improved using trap colors different to black [31,32]. We used a black cross-vane trap as Park et al. [33] reported the high efficacy of black in attracting *M. saltuarius*.

**Figure 1:**
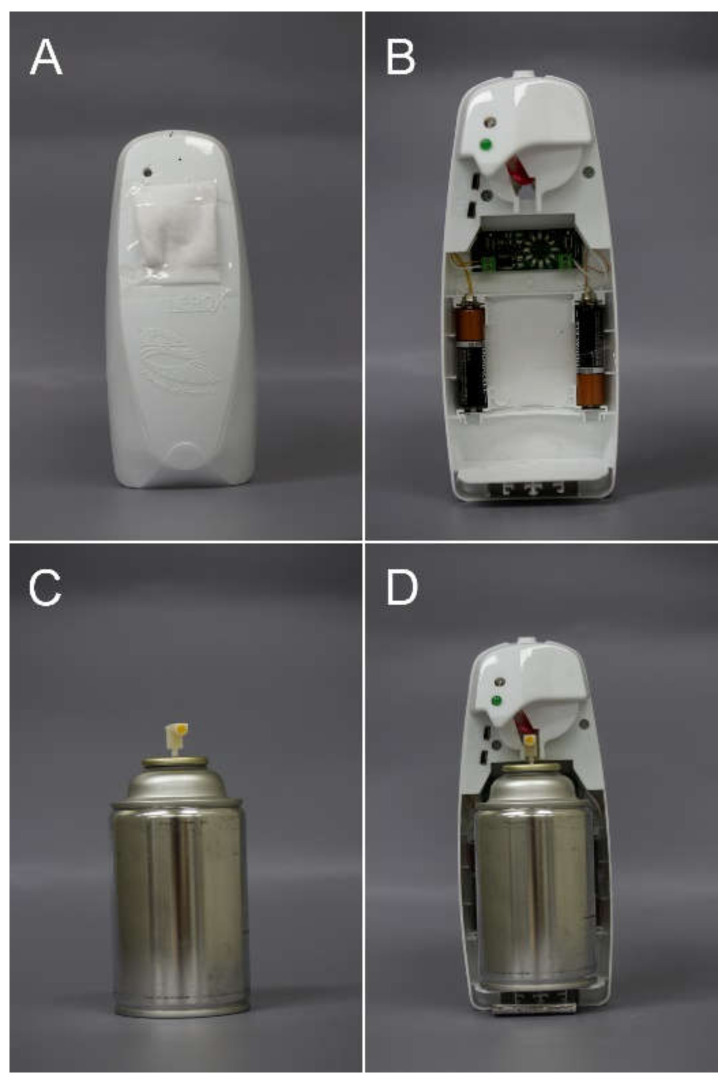
The spray devices used in the experiments. (**A**) The spray holder inside which the canister was fixed, (**B**) the spray holder with an electronic, controlled timer, (**C**) the canister, and (**D**) inside view of the spray device with the canister.

The spray dispenser consisted of a canister and a spray holder. The canister contained *M. saltuarius* PA, which was a blend of ethanol, monochamol, α-pinene, and ipsenol in a ratio of 1000:1:1:1.6. Monochamol was synthesized following a previous report [34]. The ethanol and α-pinene were purchased from Alfa Aesar (Heysham, UK), and the ipsenol was obtained from Bedoukian Research Inc. (Danbury, CT, USA). These components (PA, 90 g) were sealed in a canister (269 mL, Figure 1A) with a propellant (butane:propane = 7:3). The canister was fixed in a spray holder (Figure 1B; Glade, Johnson Korea Inc., Seoul, Korea), which controlled the spray interval. An electronic, controlled timer (Figure 1C; SM Biovision, Jinju, Korea) was installed in the spray holder to control the spray interval. To evaluate the releasing amount of PA per emission, we collected the aerosol, which was emitted 10 times from the canister in an empty glass, and weighed. This was performed six times. The devices released 30.62 ± 0.45 mg of PA per emission. To evaluate the ratio of PA in each emission, the spray solution emitted from the canister was analyzed using gas chromatography-mass spectrometry (GC-MS). GC-MS analysis was performed on a 7890A GC instrument coupled with a 5975C mass spectrometer (Agilent Technologies, Santa Clara, CA, USA) equipped with an HP-Innowax column (30 m × 0.25 mm i.d., 0.25 μm film thickness; J&W Scientific, Folsom, CA, USA). The oven temperature program was set to 40 °C for 1 min, a ramp up at 6 °C/min increase until 250 °C, and a hold at 250 °C for 4 min. The ratio of ethanol:monochamol:α-pinene:ipsenol was 1000:1.09 ± 0.15:1.51 ± 0.27:1.65 ± 0.25, which was similar to 1000:1:1:1.6.

#### 2.2.2. Effect of PA Concentration on Attraction (Emission Interval 8, 15, 30 min)

To evaluate the effect of the emission interval (a proxy for the concentration of PA), the emission interval was set at 8, 15, or 30 min each day, which was equivalent to 7.5 times (ca. 230 mg of PA/h), 4 times (ca. 31 mg of PA/h), or twice (ca. 61 mg of PA/h) per hour. The experiment used a randomized complete block (RCB) design with three treatments and five replicates. The spray devices were attached to cross-vane traps (AlphaScents, Portland, OR, USA) and hung on pine trees at a height of 1.0 m (bottom of trap to ground). Traps were separated by a distance of 10–15 m, and each block was at least 50 m apart. Beetles were collected from the traps every week.

#### 2.2.3. The Diel Rhythm of Responses to Aggregation PA

To determine the diel rhythm of responses to PA, the PA spray time was divided into 05:00–11:00 (time period A), 11:00–17:00 (time period B), 17:00–23:00 (time period C), and 23:00–05:00 (time period D). As a control, a 24-h spray time was set (24 h). The same experimental design as above was used. The emission interval was set at 15 min. Beetles were collected from the traps and sexed according to the morphological characteristics.

### 2.3. Statistical Analysis

The number of beetles caught and the proportion of beetles captured in each time period were tested using linear mixed models (LMMs). The time zone was considered as a fixed effect, whereas block was considered as a random effect. The means were compared and evaluated by the Tukey–Kramer honestly significant difference (HSD) test at the 0.05 level. Thus, we performed LMMs using the ‘lmer’ and ‘glmer’ function in the ‘lme4’ package and ‘Anova’ function in the ‘car’ package. The least square mean was used for multiple comparisons using the ‘glht’ function in the ‘multcomp’ package. All statistical analyses were performed with R v.3.5.1 [35]. The mean (+standard error [SE]) values of untransformed data are reported.

## 3. Results

### 3.1. Effect of PA Concentration on Attraction

**Figure 2:**
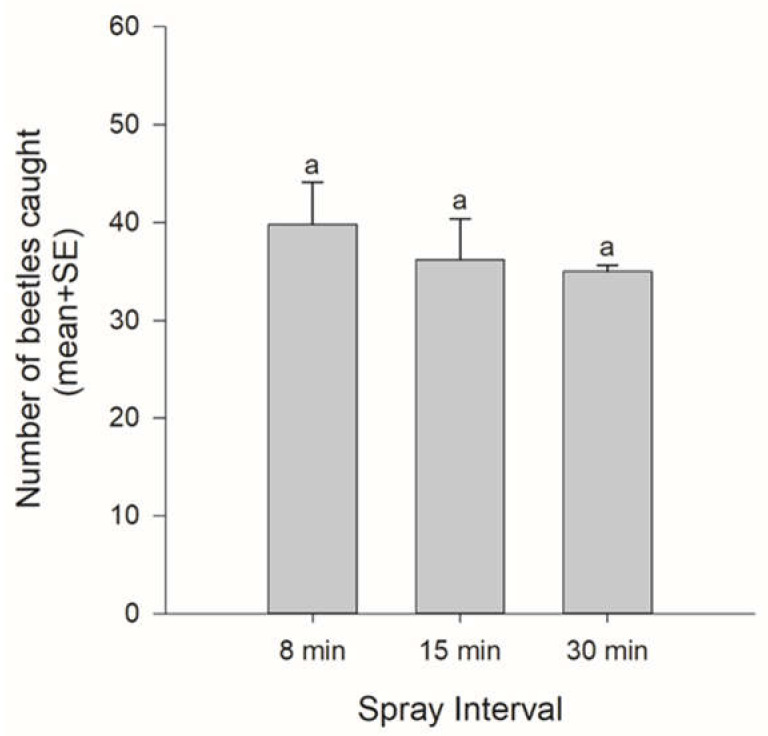
The numbers of beetles caught according to spray intervals (a proxy for the concentration of PA). Bars with the same letters were not significantly different (Tukey–Kramer HSD test, *p* > 0.05).

By altering the emission interval, we altered the concentration of PA emitted at the test sites. Only *M. saltuarius* was caught. The number of caught beetles tended to decrease as the emission interval increased. However, there was no significant difference (spray interval: *F* = 0.546, df = 2, *p* = 0.600) (Figure 2).

### 3.2. The Diel Rhythm of Response to Aggregation PA

**Figure 3:**
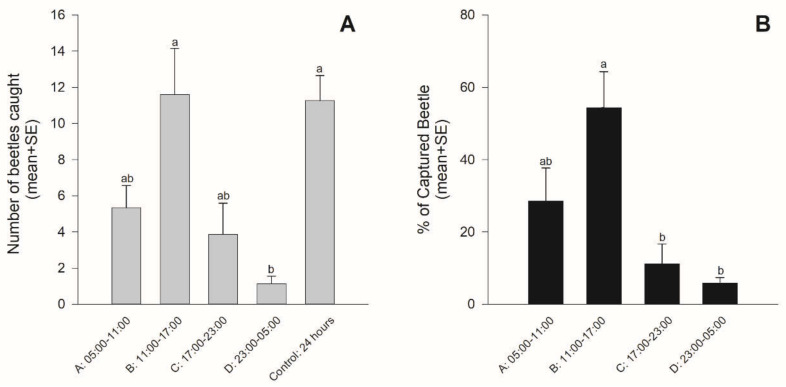
Mean (+SE) numbers of beetles captured in each time period and during the 24-h spray (**A**) and the proportion of beetles captured in each time period (**B**). Bars with the same letters were not significantly different (*p* > 0.05).

**Figure 4:**
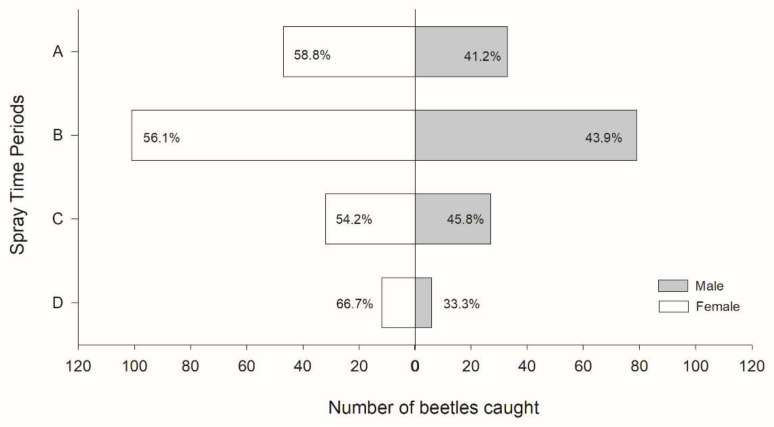
Mean numbers of beetles by sex in each spray time period. The number represents the proportion at each spray time period. Spray time period A: 05:00–11:00, B: 11:00–17:00, C: 17:00–23:00, and D: 23:00–05:00. No significant differences were observed between sexes.

Beetles were caught throughout the day (Figure 3). However, there were differences among the time periods (χ^2^ = 26.63, *df* = 4, *p* < 0.001) (Figure 3A). The number of beetles caught in time period B (1100–1700) was almost the same as that caught during the 24-h spray. Over 90% were caught in time periods A, B, and C, and less than 10% were caught in time period D (23:00–05:00) (Figure 3B). A significantly greater percentage of beetles were caught in time period B than in time periods C and D (χ^2^ = 26.499, *df* = 3, *p* < 0.001). When analyzed separately, there were no differences in the numbers of male and female beetles caught in each time period (time period A (05:00–11:00): χ^2^ = 2.45, *p* = 0.117, time period B (11:00–17:00): χ^2^ = 2.70, *p* = 0.101, time period C (17:00–23:00): χ^2^ = 0.42, *p* = 0.515, and time period D (23:00–05:00): χ^2^ = 2.04, *p* = 0.153) (Figure 4).

## 4. Discussion

Aerosol dispensers such as the Metered Semiochemical Timed Release System (MSTRS^TM^) [36,37] and Isomate^®^ CM MIST (MIST) Aerosol dispenser [38] have been used to study mating disruption or monitoring lepidopteran pests [39,40]. The merits of aerosol dispensers are that the emission ratio is conserved and the emission amount is easily changed by setting the emission interval. The spraying device used in this study also conserved the emission ratio of PA, and the emission amount was easily changed. This is the first example of using an aerosol dispenser to monitor the diel rhythm of coleopteran pests.

A previous study aimed to assess diel flight activity patterns of longhorn beetles either by exploiting timer traps, an instrument that rotates trap jars at programmable intervals [41,42], or manually removing trapped beetles from baited traps at certain time intervals [30]. Both approaches have some cons: timer traps are relatively costly, while manually removing the trapped individuals is labor-intensive. In our study, a spray dispenser equipped with an electronic, controlled timer was used. This was much cheaper than the timer trap and less labor is needed. The spay dispenser can open new scenarios in this field of research.

To test the effect of the concentration of PA on attraction, PA (monochamol, a-pinene, and ipsenol) was emitted at concentrations of 0.97, 0.52, and 0.26 mg per hour, and ethanol was emitted at 228 mg, 120 mg, and 60 mg per hour by using spraying intervals of 8, 15, and 30 min, respectively. The results showed that 0.26 mg/h of PA and 60 mg/h of ethanol were sufficient to attract beetles. Lee et al. [24] reported that the release rate of PA (monochamol, a-pinene, and ipsenol) from polyethylene sachets was 6.63 mg/h and that of ethanol was 2.2 mg/h. In our experiments and a previous report by Kim et al. [25], there was no significant difference in attraction when the sexes were analyzed separately (Figure 4). However, Lee et al. [24] reported that only pheromones and a PA mixture, with the same composition as that in this study, attracted more females than males. This difference may be due to different attractant composition ratios or different pine tree compositions in the tested forests.

Light is one of the factors that regulates flight activity [43]. Coleoptera comprise not only diurnal and nocturnal species [19,30,41,42], but also crepuscular species [44]. Therefore, to understand the diel rhythm of the response to PA, we divided the day into four time periods: time period A, 05:00–11:00 (included dawn), time period B, 11:00–17:00 (the highest temperatures of the day), time period C, 17:00–23:00 (included dusk), and time period D, 23:00–05:00 (nighttime). The results from the 8-h spray interval PA experiments conducted for six weeks showed that the response to PA peaked in time period B and declined at night (time periods C and D). This could lead to the conclusion that the flight activity of *M. saltuarius* in response to PA is diurnal. This is supported by a previous report that found that feeding by *M. saltuarius* mainly occurred from 09:00–18:00, and mating activity occurred at night [13].

The aggregation pheromone of *M. saltuarius* is produced throughout the day, and larger quantities are emitted during the day than at night, although the difference was not significant [33]. Considering the above results and our results, the diel activity of *M. saltuarius* is not associated with the production of the aggregation pheromone. *M. alternatus* emitted significantly more aggregation pheromone at night than during the day [33]. The diel activity of *M. alternatus* has been reported, and it is primarily active from 2000 to 0500. Mating, oviposition, and movement occur at night [17,19]; however, results on feeding activity are controversial. Nishimura [17] reported that feeding activity of *M. alternatus* mainly occurred at night from 2000–0500, while Fauziah et al. [16] reported feeding activity in the morning and afternoon hours from 08:00–18:00. Therefore, the diel activity of *M. alternatus* in response to PA remains to be investigated. A study in *Monochamus scutellatus scutellatus*, which produces the aggregation pheromone monochamol, showed that a diel rhythm was not associated with the production of the male-produced aggregation pheromone [30]. *M. s. scutellatus* produces its aggregation pheromone throughout the day, and responses to the aggregation pheromone mainly occurred from 06:00–14:00.

The study sites were endemic for both *M. saltuarius* and *M. alternatus*. Therefore, we expected both species to be captured. However, in our study, *M. alternatus* was not captured in the traps. This could be explained by the temporal difference in emergence and the capture efficiency of PA. *M. saltuarius* emerges from early May to early June [12], while *M. alternatus* emerges from late May to late June [45]. The capture efficiency of PA for *M. alternatus* was lower than that for *M. saltuarius* [24,46].

## 5. Conclusions

Our study revealed that the flight activity of *M. saltuarius* in response to aggregation PA was diurnal. These results improve the understanding of the behavioral biology of *M. saltuarius*, allowing the development of pest management strategies to prevent the spread of PWN and control its vector.

## Supplementary Materials

The following are available online at https://www.mdpi.com/article/10.3390/insects12050441/s1, Figure S1: Sum of beetles caught in each treatment at site A and site B through the experimental periods. Site A: Seongpyeong (36°39′27″ N, 128°24′01″ E), Yecheon, Gyeongsangbuk-do, Treatment: spray interval; Site B: Susim (36°39′23″ N, 128°24′00″ E), Yecheon, Gyeongsangbuk-do, Treatement: spray period.

## References

[1] Zhao B., Futai K., Sutherland J.R., Takeuchi Y. (2008). Pine Wilt Disease.

[2] Sato H., Sakuyama T., Kobayashi M. (1987). Transmission of *Bursaphelenchus xylophilus* (STEINER et BUHRER) NICKLE (Nematoda, Aphelenchoididae) by *Monochamus saltuarius* (GEBLER) (Coleoptera, Cerambycidae). J. Jpn. For. Soc..

[3] Zhao B.G., Zhao B., Futai K., Sutherland J.R., Takeuchi Y. (2008). Pine wilt disease in China. Pine Wilt Disease.

[4] Shin S.-C., Zhao B., Futai K., Sutherland J.R., Takeuchi Y. (2008). Pine wilt disease in Korea. Pine Wilt Disease.

[5] Pajares J.A., Alvarez G., Ibeas F., Gallego D., Hall D.R., Farman D.I. (2010). Identification and field activity of a male-produced aggregation pheromone in the pine sawyer beetle, *Monochamus galloprovincialis*. J. Chem. Ecol..

[6] Korea Forest Research Institute (2007). Annual Report of Monitoring for Forest Insect Pest and Diseases in Korea.

[7] Potenko V.V., Velikov A.V. (1998). Genetic diversity and differentiation of natural populations of *Pinus koraiensis* (Sieb. et Zucc.) in Russia. Silvae Genet..

[8] Li M., Li H., Sheng R.C., Sun H., Sun S.H., Chen F.M. (2020). The first record of *Monochamus saltuarius* (Coleoptera; Cerambycidae) as vector of *Bursaphelenchus xylophilus* and its new potential hosts in China. Insects.

[9] Pan L., Li Y., Cui R., Liu Z., Zhang X. (2020). *Monochamus saltuarius* endangers *Pinus tabuliformis* Carr. and Carries *Bursaphelenchus xylophilus* (Steiner and Buhrer) in China. Forests.

[10] Akbulut S., Stamps W.T. (2012). Insect vectors of the pinewood nematode: A review of the biology and ecology of *Monochamus* species. For. Pathol..

[11] Han J.-H., Kim H.K., Kang W.J., Kim G.-H. (2016). Feeding and oviposition preference of the Sakhalin pine sawyer *Monochamus saltuarius* (Coleoptera: Cerambycidae) for various tree species. Entomol. Res..

[12] Han J.-H., Yoon C., Shin S.-C., Kim G.-H. (2007). Seasonal occurrence and morphological measurements of pine sawyer, *Monochamus saltuarius* adults (Coleoptera: Cerambycidae). J. Asia Pac. Entomol..

[13] Kim M.-K., Han J.-H., Kim Y.-J., Yoon C., Kim G.-H. (2006). Mating behavior of pine sawyer, *Monochamus saltuarius* Gebler (Coleoptera: Cerambycidae). J. Asia Pac. Entomol..

[14] Kwon T.-S., Lim J.-H., Shin S.-J., Kwon Y.-D., Son S.-K., Lee K.-Y., Kim Y.-T., Park J.-W., Shin C.-H., Ryu S.-B. (2006). Distribution patterns of *Monochamus alternatus* and *M. salturarius* (Coleoptera: Cerambycidae) in Korea. J. Korean For. Soc..

[15] Kim G.-H., Takabayashi J., Takahashi S., Tabata K. (1992). Function of pheromones in mating behavior of the Japanese pine sawyer beetle, *Monochamus alternatus* HOPE. Appl. Entomol. Zool..

[16] Fauzia M.K., Hidaka T., Tabata K. (1987). The reproductive behavior of *Monochamus alternatus* Hope (Coleoptera: Cerambycidae). Appl. Entomol. Zool..

[17] Nishimura M. (1973). Daily observation on behaviors of Japanese pine sawyer adult, *Monochamus alternatus* Hope. J. Jpn. For. Soc..

[18] Taniwaki T., Okitsu M., Kishi Y. (2004). Diurnal emergence of *Monochamus alternatus* Hope (Coleoptera: Cerambycidae) from pine logs. J. Jpn. For. Soc..

[19] Kim D.S., Lee S.M., Kim C.S., Lee D.W., Park C.G. (2011). Movement of *Monochamus alternatus* Hope (Coleoptera: Cerambycidae) adults among young black pine trees in a screen cage. Kor. J. Appl. Entomol..

[20] Kwon H.J., Jung J.-K., Jung C., Han H., Koh S.-H. (2018). Dispersal capacity of *Monochamus saltuarius* on flight mills. Entomol. Exp. Appl..

[21] Teale S.A., Wickham J.D., Zhang F., Chen Y., Wei X., Hanks L.M., Millar J.G. (2011). A male-produced aggregation pheromone of *Monochamus alternatus* (Coleoptera: Cerambycidae), a major vector of pine wood nematode. J. Econ. Entomol..

[22] Allison J.D., McKenney J.L., Millar J.G., McElfresh J.S., Mitchell R.F., Hanks L.M. (2012). Response of the woodborers *Monochamus carolinensis* and *Monochamus titillator* (Coleoptera: Cerambycidae) to known cerambycid pheromones in the presence and absence of the host plant volatile a-pinene. Environ. Entomol..

[23] Pajares J.A., Álvarez G., Hall D.R.H., Douglas P., Centeno F., Ibarra N., Schroeder M., Teale S.A., Wang Z., Yan S. (2013). 2-(Undecyloxy)-ethanol is a major component of the male-produced aggregation pheromone of *Monochamus sutor*. Entomol. Exp. Appl..

[24] Lee H.-R., Lee S.-C., Lee D.H., Choi W.-S., Jung C.-S., Jeon J.-H., Kim J.-E., Park I.-K. (2017). Identification of the aggregation-sex pheromone produced by male *Monochamus saltuarius*, a major insect vector of the pine wood nematode. J. Chem. Ecol..

[25] Kim J., Lee S.-M., Jung Y.H., Kwon Y.-D., Kim D.-S., Lee D.W., Park C.G. (2016). Field evaluation on the synergistic attractiveness of 2-(1-undecyloxy)-1-ethanol and ipsenol to *Monochamus saltuarius*. Entomol. Res..

[26] Ma T., Shi X., Shen J., Wang C., Zhang S., Lu X., Sun Z., Chen X., Wang C., Xie W. (2018). Field evaluation of commercial attractants and trap placement for monitoring pine sawyer beetle, *Monochamus alternatus* (Coleoptera: Cerambycidae) in Guangdong, China. J. Econ. Entomol..

[27] Miller D.R., Allison J.D., Crowe C.M., Dickinson D.M., Eglitis A., Hofstetter R.W., Munson A.S., Poland T.M., Reid L.S., Steed B.E. (2016). Pine sawyers (Coleoptera: Cerambycidae) attracted to α-pinene, monochamol, and ipsenol in North America. J. Econ. Entomol..

[28] Schroeder M. (2019). Trapping strategy for *Monochamus sutor* and *Monochamus galloprovincialis*: Potential vectors of the pine wood nematode in Scandinavia. Agric. For. Entomol..

[29] Boone C.K., Sweeney J., Silk P., Hughes C., Webster R.P., Stephen F., Maclauchlan L., Bentz B., Drumont A., Zhao B. (2018). Monochamus species from different continents can be effectively detected with the same trapping protocol. J. Pest Sci..

[30] Skabeikis D.D., Teale S.A., Fierke M.K. (2016). Diel rhythms in *Monochamus* (Coleoptera: Cerambycidae): Production of and response to a male-produced aggregation pheromone. Environ. Entomol..

[31] Cavaletto G., Faccoli M., Marini L., Spaethe J., Giannone F., Moino S., Rassati D. (2020). Exploiting trap color to improve surveys of longhorn beetles. J. Pest Sci..

[32] Lyu F., Hai X.-X., Wang Z.-G. (2021). Green-colored paperboard enhances the Asian longhorned beetle response to host plant odor cues. J. Pest Sci..

[33] Park I.K., Lee S.C., Hur M.J., Kwon J.H. (2018). Research on Improvement the Attractivenss of Aggregation-Sex Pheromone and Analysis of Behaviour Response.

[34] Kim J., Lee S.-M., Park C.G. (2016). *Bursaphelenchus xylophilus* is killed by homologues of 2-(1-undecyloxy)-1-ethanol. Sci. Rep..

[35] Team R.C. (2018). R: A Language and Enviroment for Statistical Computing.

[36] Baker T.C., Dittl T., Mafra-Neto A. (1997). Disruption of sex pheromone communication in the blackheaded fireworm in Wisconsin cranberry marshes by using MSTRS™ devices. J. Agric. Entomol..

[37] Baker T.C., Mafra-Neto A., Dittl T., Rice M.E., Witzgall P., Minks A.K. (1997). A novel controlled-release device for disrupting sex pheromone communication in moths. Technology Transfer in Mating Disruption IOBC wprs Bull.

[38] McGhee P.S., Miller J.R., Thomson D.R., Gut L.J. (2016). Optimizing aerosol dispensers for mating dsruption of codling moth, *Cydia pomonella* L.. J. Chem. Ecol..

[39] Burks C.S., Thomson D.R. (2020). Factors affecting disruption of navel orangeworm (Lepidoptera: Pyralidae) using aerosol dispensers. J. Econ. Entomol..

[40] Stelinski L., Gut L., Haas M., McGhee P., Epstein D. (2007). Evaluation of aerosol devices for simultaneous disruption of sex pheromone communication in *Cydia pomonella* and *Grapholita molesta* (Lepidoptera: Tortricidae). J. Pest Sci..

[41] Mitchell R.F., Reagel P.F., Wong J.C.H., Meier L.R., Silva W.D., Mongold-Diers J.A., Millar J.G., Hanks L.M. (2015). Cerambycid beetle species with similar pheromones are segregated by phenology and minor pheromone components. J. Chem. Ecol..

[42] Rassati D., Marchioro M., Flaherty L., Poloni R., Edwards S., Faccoli M., Sweeney J. (2020). Response of native and exotic longhorn beetles to common pheromone components provides partial support for the pheromone-free space hypothesis. Insect Sci..

[43] Landin B.-O. (1968). The diel flight activity of dung-beetles (Coleoptera Scarabaeidae). A study of the influence of environmental factors, with particular reference to the light. Opusc. Entomol. Suppl..

[44] Feer F., Pincebourde S. (2005). Diel flight activity and ecological segregation within an assemblage of tropical forest dung and carrion beetles. J. Trop. Ecol..

[45] Park C.G., Kim D.S., Lee S.M., Moon Y.S., Jin C.Y., Kim D.-S. (2014). A forecasting model for the adult emergence of overwintered *Monochamus alternatus* (Coleoptera: Cerambycidae) larvae based on degree-days in Korea. Appl. Entomol. Zool..

[46] Lee H.-R., Lee S.-C., Lee D.H., Jung M., Kwon J.-H., Huh M.-J., Kim D.S., Lee J.-E., Park I.-K. (2018). Identification of aggregation-sex pheromone of the Korean *Monochamus alternatus* (Coleoptera: Cerambycidae) population, the main vector of pine wood nematode. J. Econ. Entomol..

