# Supplementary material for: Diel Rhythmicity of Field Responses to Synthetic Pheromone Lures in the Pine Sawyer Monochamus saltuarius
Source: Insects. 2021 May 12;12(5):441. doi: 10.3390/insects12050441 (PMC8151719; doi:10.3390/insects12050441)
Supplement: Supplementary file 1 [file insects-12-00441-s001.zip › insects-1195297-supplementary.pdf]

Supplemental Information

Diel rhythmicity of field responses to synthetic pheromone lures in the pine sawyer *Monochamus saltuarius*

Junheon Kim[1,*], Young Hak Jung [2], Sang-Myeong Lee [2]

[1] Forest Insect Pests and Diseases Division, National Institute of Forest Science, Seoul 02455, Republic of Korea; junheonkim@korea.kr
[2] SM Biovision Co., Jinju, Gyeongnam, Republic of Korea; gkr315@naver.com; lsm1918@hanmail.net
* Correspondence: junheonkim@korea.kr; Tel.: +82-2-961-2672

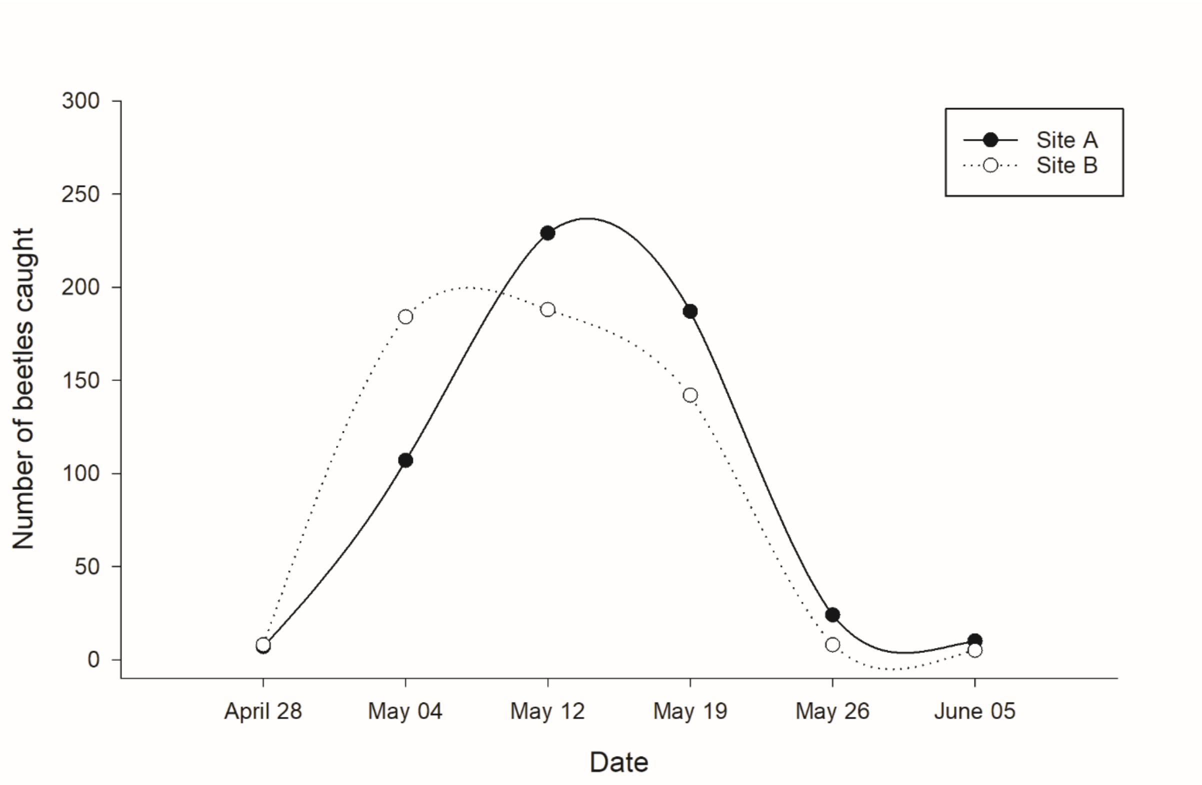

Figure S1. Sum of beetles caught in each treatment at site A and site B through the experimental periods.

Site A: Seongpyeong (36°39′27″N, 128°24′01″E), Yecheon, Gyeongsangbuk-do, Treatment : spray interval

Site B: Susim (36°39′23″N, 128°24′00″E), Yecheon, Gyeongsangbuk-do, Treatement: spray period
